# Are patients' judgments of health status really different from the general population?

**DOI:** 10.1186/1477-7525-9-31

**Published:** 2011-05-11

**Authors:** Paul FM Krabbe, Noor Tromp, Theo JM Ruers, Piet LCM van Riel

**Affiliations:** 1 Department of Epidemiology, Unit Health Technology Assessment, University Medical Center Groningen, University of Groningen, Groningen, The Netherlands; 2 Radboud University Nijmegen Medical Centre, International Center for Health Systems Research and Education, (NICHE), Department of Primary and Community Care, P.O. Box 9101 6500 HB Nijmegen, The Netherlands; 3 Antoni van Leeuwenhoek Hospital, Department of Surgery, Plesmanlaan 121, 1066 CX Amsterdam, The Netherlands; 4 Radboud University Nijmegen Medical Centre, Department of Rheumatology, P.O. Box 9101, 6500 HB Nijmegen, The Netherlands

## Abstract

**Background:**

Many studies have found discrepancies in valuations for health states between the general population (healthy people) and people who actually experience illness (patients). Such differences may be explained by referring to various cognitive mechanisms. However, more likely most of these observed differences may be attributable to the methods used to measure these health states. We explored in an experimental setting whether such discrepancies in values for health states exist. It was hypothesized that the more the measurement strategy was incorporated in measurement theory, the more similar the responses of patients and healthy people would be.

**Methods:**

A sample of the general population and two patient groups (cancer, rheumatoid arthritis) were included. All three study groups judged the same 17 hypothetical EQ-5D health states, each state comprising the same five health domains. The patients did not know that apart from these 17 states their own health status was also included in the set of states they were assessing. Three different measurement strategies were applied: 1) ranking of the health states; 2) placing all the health states simultaneously on a visual analogue scale (VAS); 3) separately assessing the health states with the time trade-off (TTO) technique. Regression analyses were performed to determine whether differences in the VAS and TTO can be ascribed to specific health domains. In addition, effect of being member of one of the two patient groups and the effect of the assessment of the patients' own health status was analyzed.

**Results:**

Except for some moderate divergence, no differences were found between patients and healthy people for the ranking task or for the VAS. For the time trade-off technique, however, large differences were observed between patients and healthy people. The regression analyses for the effect of belonging to one of the patient groups and the effect of the value assigned to the patients' own health state showed that only for the TTO these patient-specific parameters did offer some additional information in explaining the 17 hypothetical EQ-5D states.

**Conclusions:**

Patients' assessment of health states is similar to that of the general population when the judgments are made under conditions that are defended by modern measurement theory.

## Introduction

Health status or health-related quality of life (HRQoL) can be measured by two distinct methods. The first produces descriptive profile measures encompassing multiple health domains. Examples of descriptive health measurement instruments are the SF-36 and, in the field of cancer, the EORTC QLQ C-30. In the second method, overall HRQoL is quantified as a single metric figure. The latter is referred to as a value-based methodology or index approach. Several different techniques (e.g., standard gamble, time trade-off, visual analogue scale, discrete choice models) are used to derive such values (variously called utilities, preferences, strength of preference, index, or weights).

In science it is essential to focus on two fundamental measurement properties: reliability and validity. Both are important, the latter even crucial; valid measurement implies that health outcome measures are meaningful and measure what they are supposed to measure. Preferably, health outcome measures should also be suited to computational procedures and statistical testing. For that reason, informative (i.e., metric) outcome measures should be at least at the interval level. This means that measures should lie on a unidimensional continuous scale, whereby the differences between values reflect true differences (i.e., if a patient's score increases from 40 to 60, this increase is the same as from 70 to 90). Such measures can provide vital information for health outcomes research, economic evaluations, clinical monitoring, and disease modeling studies.

Conventionally, the values for different health states used in economic evaluations are derived from a representative community sample [1]. Subjects who value the hypothetical health states need not be familiar with specific illnesses. However, it is reasonable to assume that in many situations healthy people may be inadequately informed or lack good imagination to make an appropriate judgment about the impact of (severe) health states. For this and other reasons it is not surprising that the field of HRQoL research is engaged in debate about which values are more valid. Many authors assert that individuals are the best judges of their own health status. Therefore, in a health-care context, it is the patient's judgment that should be elicited, not that of a sample of unaffected members of the general population.

Several investigators have noted that patients who have experienced a particular health state often assign higher values to their own state than do members of the general population for the same state [2-4]. A number of studies report discrepancies in the values obtained from patients and the general population [5,6]. Nonetheless, a recent meta-analysis demonstrates the absence of systematic differences [7]. Other studies conclude that people attach different values to hypothetical health states, depending on their own health condition [8,9].

Prominent though not necessarily mutually exclusive explanations for such discrepancies include 'adaptation mechanisms' [10-12], 'response shift' [13,14], 'cognitive dissonance' [15,16], and the implications of 'prospect theory' [17]. The most frequent proposition holds that the difference is largely related to the level of 'experience' of the assessor, implying that adaptation (and therefore redefinition of what is good health) comes with experience.

However, most of these observations are not based on direct comparisons of patients' valuations with those of the general population. Furthermore, many of the patients in these studies were not confronted with a variety of health states, ranging from mild to severe, but were only assessing a few disease-related or treatment-related health-state outcomes [18-22]. Moreover, in most of these studies health states were assessed in a *monadic *approach. This means that health states were assesses state-by-state. Yet, discrimination is a basic operation of judgment and of generating knowledge which explains that the core activity of the quantification of subjective phenomena in measurement theory is to compare two or more entities in such a way that the data yields compelling information [23-25]. Consequently, much of the observed difference between patients' valuations of their own health state and the values assigned to health states by healthy people may be attributed to the applied measurement framework.

Our objective was twofold i) to explore in an experimental way whether discrepancies in values for health states exist between the general population and people who actually experience specific illness (patients); ii), whether such discrepancies depends on the applied measurement approach. It was hypothesized that the more measurement strategies were supported by measurement theory, the more similar the responses of patients and healthy people would be.

## Methods

### Subjects

Two different patient groups from the Radboud University Nijmegen Medical Centre (Netherlands) participated in the study, which was approved by the Central Committee on Research Involving Human Subjects (region Arnhem-Nijmegen). We deliberately selected two patients groups that were quite different to create a contrast in our experimental study. (For that reason background characteristics are expected to be different and no statistical adjustments are made for them.) One group included patients that were diagnosed with cancer within a time frame of 4-6 weeks before they participated in the study. Since all cancer patients were planned to undergo surgery, meaning that the stage of their disease was comparable, differences in life expectancy were limited. The other group consisted of chronically ill patients living with the symptoms of rheumatoid arthritis (RA) for at least 3 years. All patients were approached in the clinic by their physician. Informed consent was obtained by the physician (TJMR, PLCMR) and interviewer. Representative general population (healthy people) data were obtained from a Dutch valuation study in which the principal investigator (PFMK) participated [26]. In this study with healthy people exactly the same study protocol was followed as in the study with the patients, which guaranties that the measurement conditions were similar in the two study groups. Only the general population group received a gift voucher worth 20 euros for participation.

### Health states

The EuroQol-5D (EQ-5D) classification describes health status according to five attributes: mobility; self-care; usual activities; pain/discomfort and anxiety/depression. Each attribute has three levels: level 1 'no problems'; level 2 'some problems'; and level 3 'severe problems' [27]. Health-state descriptions are constructed by taking one level for each attribute, thus defining 243 (3^5^) distinct health states ('11111' represents the best health state). A fix set of 17 EQ-5D health-state descriptions were selected. This set comprised 5 very mild, 4 mild, 4 moderate, and 3 severe states and also state '33333'. These states were selected on the grounds of the Dutch-based EuroQol tariff design developed in 2006 [26]. All EQ-5D health-state descriptions were printed on cards. Respondents were instructed that for a health state to be considered unchangeable, it had to persist for ten years and be followed by dead.

### Judgmental tasks

The study protocol was administered face-to-face by a trained interviewer (NT) at the homes of the patients. All patients (as well as the general population sample) assessed the same set of 17 EQ-5D health states by performing the same three judgmental tasks in exactly the same way. Two weeks in advance (postal), to record their current health state all patients described their own health status using the standard EQ-5D classification. Additionally, each patient unknowingly assessed his or her own health status in all three judgmental tasks as the own EQ-5D health-state description had been incorporated in the set. Instructions were repeated until the interviewer judged that the respondent understood the task. For each judgmental tasks all states were presented in random order to control for potential biases due to presentation order or respondent fatigue.

#### Ranking

The first and most elementary judgmental task consisted of ranking the 17 EQ-5D health states, supplemented with the patient's own EQ-5D description, 'dead', and state '11111.' (note: 'dead' and '11111' were not judged in the time trade-off task. See below). This task can be considered a step-by-step paired comparison task, featuring a distinct comparative or discrimination mechanism [28]. Each patient ranked these same 20 health states by putting the card with the 'best' health state on top and the 'worst' at the bottom.

#### Multi-item visual analogue scale (VAS)

After the ranking task, patients were instructed to place the 20 cards on the standard EuroQol (multi-item) VAS (EQ-VAS). The standard EQ-VAS consists of a 20 cm thermometer-like vertical line with end-points (anchors) of 100 for the 'best imaginable health state' and 0 for the 'worst imaginable health state'. The respondent rates the desirability of each health state by placing its card at some point along the scale. This VAS exercise employed a bisection method [29]. First, the state ranked 'best' was located on the VAS, followed by the one ranked 'worst', and then the state closest to lying half-way on the scale (i.e., between the two extreme states already in place). Subsequently, two states were located between the half-way state and the two extreme states. Finally, all residual states were located simultaneously on the VAS. The instruction was to locate the cards in such way that the intervals between the positions of the health states corresponded with their perceived differences. A critical assumption underlying the multi-item VAS task is that respondents are not only implicitly comparing health states and making decisions about which ones are preferable (ranking), but are also adjusting the distances between the array of states in such a way that the positions reflect the differences in preferences for these states.

#### Time trade-off (TTO)

The VAS valuation task was followed by the TTO valuation of the same set of EQ-5D states, except for state '11111' and 'dead'. These two states cannot be directly valued, as in TTO their values are pre-assigned to 1 and 0 respectively. TTO requires respondents to trade longevity for improved health in choices between certain prospects [30]. The TTO task was executed by a Computer Assisted Personal Interviewing (CAPI) method. Computer software integrated the TTO study protocol, scoring administration, and the visual aid. The program presented the standardized health states (including the patient's health state) in random order and replaced the classic TTO boards of the original UK study protocol [31]. Respondents were led by a process of outward titration to select a length of time *t *in state '11111' (full health) that they regarded as equivalent to 10 years in the target state. The shorter the 'equivalent' length of time in full health, the worse the target state is. The interviewer handled each TTO session by giving instructions to the respondent and operating the software buttons.

### Analyses

Respondents were excluded if 1) fewer than 3 health states were valued, 2) all health states were given the same value, and 3) state 11111 or dead was not valued or dead > state 11111 [26]. This last exclusion criterion was only applied for the VAS. It is necessary when rescaling "raw" VAS scores to values on the 0 (dead) to 1 (full health) 'utility' scale. Rescaling (e.g., calibration) was performed at the respondent level on the basis of the observed VAS scores for the various health states, and the scores that were recorded for "dead" and "full health" (e.g., state 11111), using the following equation:

Transformation of the TTO scores was based on the standard EuroQol approach. For states regarded as better than dead, the TTO value (v) is *t*/10; for states worse than dead, values are computed as -*t*/(10 - *t*). These negative health states were subsequently bounded at minus 1 with the commonly used transformation *v' *= *v*/(1 - *v*).

Descriptive statistics were calculated for the background characteristics of the three samples. Then frequency distributions were made for the classification of the patients' health state. Mean scores and standard errors of the mean were calculated for the various assessments of the (hypothetical) health states. For the non-patient group, ranks were adjusted for the fact that this group assessed one health state less (own state) than the two patient groups. Regression analyses were performed for the VAS and TTO data to estimate the effect of the different domains, the effect of being member of one of the two patient groups, and the effect of the assessment of the patients' own EQ-5D health state. In these regression analyses we applied the standard EuroQol model which is based on variables for the 5 domains (for each domain 2 dummies expressing the step from level 1 to level 2, and the step from level 2 to level 3) extended with the N3 dummy variable. This N3 parameter is a nonmultiplicative interaction term that is frequently used in EuroQol valuation models. It allows for measuring the "extra" disutility when reporting severe (level 3) problems on at least one EQ domain.

All statistical analyses were performed with SPSS (version 17.0), the diagrams were drawn with SigmaPlot (version 11).

## Results

### Respondents

In total 75 patients were interviewed (approx. 1.5 - 2.5 hours). Of the 50 cancer patients (36 colorectal cancer, 14 breast cancer) approached for participation, 48 gave their consent (96% response). The RA patients' response rate was 75%, with 27 of the 36 patients approached consenting to participation. Reasons to refuse were 'not interested ' or 'no time'. The general population (healthy people) consists of 212 respondents. The main characteristics of the three samples are presented in Table 1.

**Table 1:** Demographic characteristics and health condition of the study populations

	Cancer patients (n = 48)	Rheumatoid Arthritis patients (n = 27)	General population (n = 212)
Gender (male, %)	58.7	34.6	50.0
			
Age (Mean, sd)	63.1 (9.7)	64.5 (9.1)	44.0 (16.3)
			
Educational level (%)			
Low	47.8	63.0	35.8
Middle	23.9	14.8	35.4
High	28.3	22.2	28.8
			
Marital Status (%)			
Single	4.3	3.7	33.2
Married/living together	84.8	74.1	53.6
Widowed	10.9	14.8	6.6
Divorced	0.0	7.4	6.6
			
Religious (%)	63.0	70.4	46.9
			
Reporting problems own health (EQ-5D, %)			
Mobility	22.9	70.4	13.3
Self-care	0.0	44.4	1.9
Usual Activities	29.2	85.2	14.2
Pain/discomfort	35.4	88.9	33.0
Anxiety/depression	25.0	14.8	13.2
			
VAS value own health state (Mean, sd)	84.1 (2.4)	60.9 (4.2)	-
TTO value own health state (Mean, sd)	0.93(0.02)	0.74 (0.09)	-

The mean ages for the cancer patients and the RA patients were similar (63.1 vs. 64.5). The patients were on average 20 years older than the general population. Overall, the RA patients had more problems on all dimensions except anxiety. For example, 70.4% of the RA patients reported mobility problems, compared with only 22.9% of the cancer group. Education levels were equally distributed in the general population, whereas for the patient groups the lowest category was over-represented. Cancer patients showed better EQ-5D classifications of their own health condition than the RA patients (Table 2). Almost 80% of the general population sample had EQ-5D health states with no complaints or only moderate complaints in one of the five health domains.

**Table 2:** Number (%) of EuroQol-5D descriptive classifications of study populations

EuroQol-5D classification	Cancer patients (n = 48)	Rheumatoid Arthritis patients (n = 27)	General population (n = 211)
11111	19 (39.6)	2 (7.4)	123 (58.3)
11112	4 (8.3)	-	8 (3.8)
11121	4 (8.3)	2 (7.4)	29 (13.7)
11211	2 (4.2)	-	4 (1.9)
11212	1 (2.1)	-	1 (0.5)
11221	2 (4.2)	3 (11.1)	
11122	-	-	7 (3.3)
11222	5 (10.4)	-	3 (1.4)
12221	-	1 (3.7)	
12223	-	-	1 (0.5)
21111	5 (10.4)	-	4 (1.9)
21112	-	-	1 (0.5)
21121	1 (2.1)	-	7 (3.3)
21122	1 (2.1)	-	1 (0.5)
21221	3 (6.3)	7 (25.9)	5 (2.4)
21222	1 (2.1)	1 (3.7)	4 (1.9)
21223	-	-	1 (0.5)
21223	-	-	2 (0.9)
22121	-	-	1 (0.5)
22221	-	4 (14.8)	1 (0.5)
22222	-	-	1 (0.5)
22231	-	1 (3.7)	-
22232	-	2 (7.4)	-
22311	-	1 (3.7)	-
22321	-	1 (3.7)	-
22331	-	1 (3.7)	-
22332	-	1 (3.7)	-

### Health state judgments

**Figure 1:**
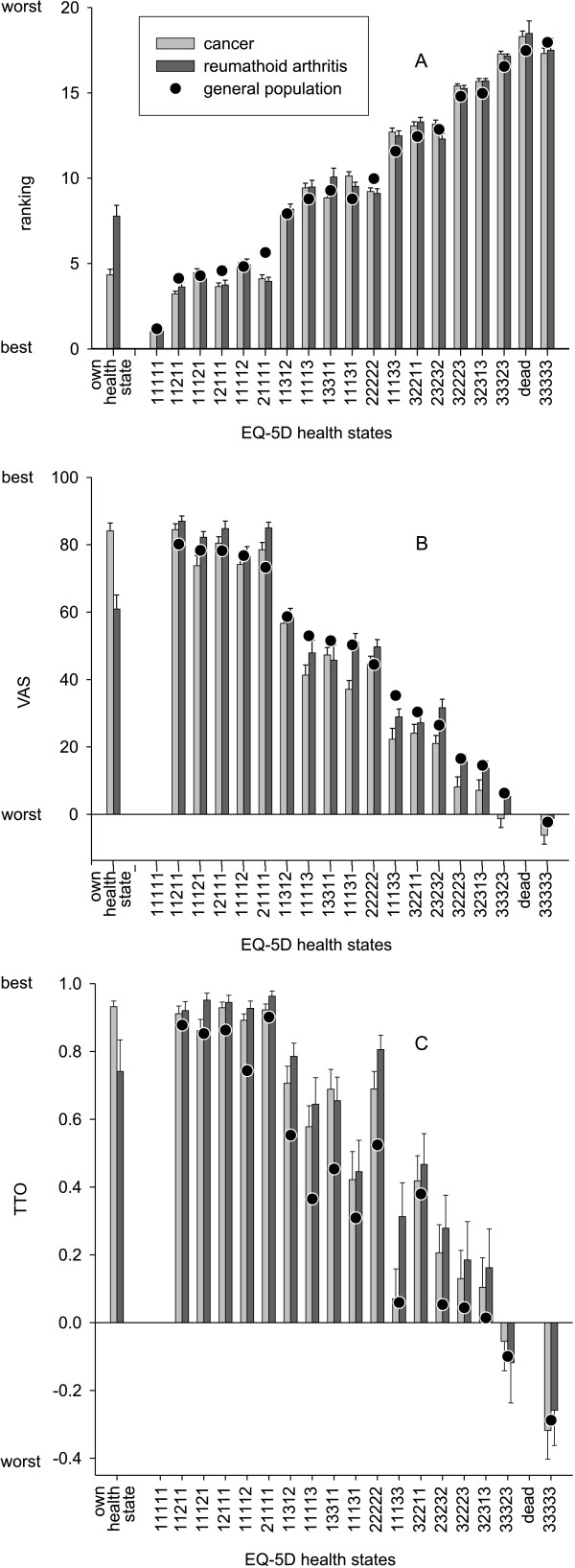
**Mean scores (added with standard error of means) of the set of EuroQol-5D health states derived by three different measurement methods (ranking, VAS, TTO) presented for the general population and for the two patient groups (For the VAS and the TTO the EuroQol-5D state '11111' is set to 1.0 and the condition 'dead' to 0.0 by definition)**.

We found almost parallel lines between the three study groups for the mean ranking scores of the assessed hypothetical health states (Figure 1A). The patients' own state was ranked as less severe than state '11312' by cancer patients and as almost comparable to this state by the RA group. It is also clear that cancer patients and RA patients ranked state '21111' (some mobility problems) as less severe than healthy people did. In the comparison of the VAS values, RA patients show a pattern closely resembling the general population (Figure 1B). For the states with only one domain at level 2, however, it seems that RA patients assign slightly higher values to these states. Compared with the general population, cancer patients seem to respond more negatively to health states associated with problems in the domains of pain/discomfort and anxiety/depression. Apart from the deviation shown by the cancer group, a gradient decline can be observed over the 17 EQ-5D states. The TTO values (Figure 1C) show higher patient values for almost all health states. Differences among the three study groups are substantially greater for the TTO data than for the rank and VAS data. Furthermore, the TTO values for the EQ-5D health states cannot be described as a gradient decline; the plot looks more like a step function.

Separate regression analyses on the VAS data for the three study groups showed that states with mobility at level 2 (some problems) were systematically assigned lower values by the general population (Table 3). This indicates that healthy people value the lack of mobility limitation as more important than the two disease groups. Furthermore, states with multiple domains with severe problems (N3 parameter) were assessed lower (-0.28) by the cancer group than by the other two groups. The proportion of explained variance (R^2^) was higher for the two patient groups (cancer: 0.76, RA: 0.83) than for the general population (0.58). An additional regression analysis showed that neither membership of one of the patient groups was an important factor to explain the valuations of 17 hypothetical EQ-5D states nor the value assigned to the patients' own health state.

**Table 3:** Coefficients (standard error) of different regression analyses on VAS values for the general population and for the two patient groups based on variables for the 5 domains (for each domain 2 dummies expressing the step from level 1 to level 2 (2), and the step from level 2 to level 3 (3))

Parameters	Coefficients				
	**Effect of EQ-5D domains**			**Additional effect of patient groups**	**Additional effect of valuation own health**
			
	**Cancer**	**RA**	**General population**	**Cancer + RA + Gen. pop**.	**Cancer + RA**

Constant	0.87 (0.01)*	0.91 (0.01)*	0.87 (0.01)*	0.88 (0.01)*	0.94 (0.02)*
Mobility (2)	-0.09 (0.02)*	-0.06 (0.02)*	-0.13 (0.01)*	-0.11 (0.01)*	-0.08 (0.02)*
Self-care (2)	-0.10 (0.02)*	-0.11 (0.02)*	-0.10 (0.01)*	-0.10 (0.01)*	-0.10 (0.02)*
Usual activities (2)	0.00 (0.02)	-0.03 (0.02)	-0.04 (0.01)*	-0.03 (0.01)*	0.01 (0.02)
Pain/discomfort (2)	-0.12 (0.02)*	-0.08 (0.02)*	-0.08 (0.01)*	-0.09 (0.01)*	-0.10 (0.01)*
Anxiety/depression (2)	-0.07 (0.02)*	-0.08 (0.02)*	-0.06 (0.01)*	-0.06 (0.01)*	-0.07 (0.02)*
Mobility (3)	-0.21 (0.03)*	-0.19 (0.03)*	-0.22 (0.02)*	-0.22 (0.01)*	-0.20 (0.02)*
Self-care (3)	-0.07 (0.03)*	-0.10 (0.03)*	-0.07 (0.02)*	-0.08 (0.01)*	-0.08 (0.02)*
Usual activities (3)	-0.02 (0.03)	-0.06 (0.03)*	-0.09 (0.02)*	-0.08 (0.01)*	0.03 (0.02)
Pain/discomfort (3)	-0.19 (0.02)*	-0.15 (0.02)*	-0.18 (0.01)*	-0.18 (0.01)*	-0.17 (0.02)*
Anxiety/depression (3)	-0.17 (0.02)*	-0.17 (0.02)*	-0.15 (0.01)*	-0.16 (0.01)*	-0.17 (0.02)*
N3	-0.28 (0.02)*	-0.24 (0.02)*	-0.17 (0.01)*	-0.20 (0.01)*	-0.26 (0.02)*
					
Cancer patients	-	-	-	-0.05 (0.01)*	-
RA patients	-	-	-	0.01 (0.01)	-
					
VAS value own state	-	-	-	-	<0.01 (0.00)
					
R^2^	0.76	0.83	0.58	0.62	0.78

*statistically significant (p _<_0.05)

Similar regression analysis on the TTO data showed that states with some problems (level 2) on the domains self-care (-0.10) and anxiety/depression (-0.13) were systematically assigned lower values by the general population (Table 4). For the two patient groups severe problems (level 3) on mobility produced lower values in comparison with the group of healthy people. For both patient groups, the coefficients for the N3 parameter (-0.12) were about half the weight of that for the general population (-0.25). The proportion of explained variance for the TTO data was lower than for the VAS data, and differences between the three study groups were less pronounced (cancer 0.45, RA 0.49, general population 0.40). The regression analyses for the effect of belonging to one of the patient groups and the effect of the value assigned to the patients' own health state showed that these patient-specific parameters did offer additional information in explaining the 17 hypothetical EQ-5D states. In particular, patients who rated themselves better in comparison with other patients rated the hypothetical health states higher. However, this effect was not expressed in the overall amount of explained variance (0.49).

**Table 4:** Coefficients (standard error) of different regression analyses on TTO values for the general population and for the two patient groups (for each domain 2 dummies expressing the step from level 1 to level 2 (2), and the step from level 2 to level 3 (3))

Parameters	Coefficients				
	**Effect of EQ-5D domains**			**Additional effect of patient groups**	**Additional effect of valuation own health**
			
	**Cancer**	**RA**	**General population**	**Cancer + RA + Gen. pop**	**Cancer + RA**

Constant	0.96 (0.03)	0.98 (0.04)	0.93 (0.02)	0.94 (0.01)*	0.78 (0.03)*
Mobility (2)	-0.04 (0.06)	-0.02 (0.07)	-0.04 (0.03)	-0.04 (0.02)*	-0.03 (0.04)
Self-care (2)	-0.03 (0.05)*	-0.02 (0.06)	-0.10 (0.03)	-0.07 (0.02)*	-0.02 (0.04)
Usual activities (2)	-0.04 (0.06)*	-0.02 (0.07)	-0.02 (0.03)	-0.04 (0.02)*	-0.03 (0.04)
Pain/discomfort (2)	-0.10 (0.04)*	-0.08 (0.05)*	-0.09 (0.02)	-0.09 (0.01)*	-0.09 (0.03)*
Anxiety/depression (2)	-0.06 (0.05)*	-0.03 (0.06)	-0.13 (0.03)	-0.11 (0.02)*	-0.05 (0.04)
Mobility (3)	-0.32 (0.07)*	-0.38 (0.08)*	-0.17 (0.04)*	-0.18 (0.02)*	-0.35 (0.05)*
Self-care (3)	-0.07 (0.06)*	-0.16 (0.07)	-0.14 (0.03)*	-0.15 (0.02)*	-0.10 (0.04)
Usual activities (3)	-0.09 (0.07)*	-0.06 (0.08)	-0.06 (0.04)	-0.07 (0.02)*	-0.08 (0.05)
Pain/discomfort (3)	-0.44 (0.05)*	-0.35 (0.06)*	-0.32 (0.03)*	-0.34 (0.02)*	-0.40 (0.04)*
Anxiety/depression (3)	-0.28 (0.05)*	-0.22 (0.06)*	-0.30 (0.03)*	-0.33 (0.02)*	-0.26 (0.04)*
N3	-0.12 (0.05)*	-0.12 (0.06)	-0.25 (0.03)	-0.21 (0.02)*	-0.11 (0.04)*
					
Cancer patient	-	-	-	0.07 (0.02)*	-
RA patient	-	-	-	0.12 (0.02)*	-
					
TTO value own state	-	-	-	-	0.22 (0.03)*
					
R^2^	0.45	0.49	0.40	0.41	0.49

*statistically significant (p < 0.05)

## Discussion

Many studies have found discrepancies in valuations for health states between the general population (healthy people) and people who actually experience illness (patients). Such differences may be explained by referring to various cognitive mechanisms. However, more likely most of these observed differences may be attributable to the approach used to measure these health states. In this study we compared different measurement strategies. One method based on the separate assessment of each health state, and two other methods that incorporated a comparative element by making judgments of at least pairs of states. Also, in contrast to many previous studies, patients did not assess a limited number of health states but agreed to judge a bundle of hypothetical health states. Such a strategy based on sets of health states better contextualizes the judgmental task for each separate health state.

For values attached to hypothetical health states, no general pattern could be detected that shows deviation between healthy people and ill people. Judgments based on ranks were rather similar for the two patient groups and the group of healthy people. In regard to the VAS and TTO methods, in which respondents are required not only to compare but also to express strength of preference, these two methods showed different values between healthy people and patients, though these differences were moderate for the VAS and large for the TTO. In addition, regression analyses showed that the own health condition seems to affect TTO valuations but not the VAS valuations.

The reduction of discrepancies between patients and the general population for the VAS may be largely due to characteristics of the judgmental (multi-item) task [32]. Other measurement methods with a comparative element have been introduced for the valuation of health states. Important methods in this area are paired comparisons [33], discrete choice analysis [34], and multidimensional scaling [35]. The popular TTO technique adopted from the field of health economics reveals far more deviation between patients and the general population. In an earlier study, the application of a basic mathematical routine also revealed deviating response behavior in health-state valuations elicited with the TTO technique [36]. It is above all the central element time that likely induce different values for different respondents in the TTO. For example, many people show unwillingness to sacrifice any life expectancy in TTO tasks. It is conceivable that the time-frame of 10 years for the TTO in this study has lead to very different value judgments between patients and the general population because the general population in our study is, on average, 20 years younger than the patients. TTO seems contaminated by an appraised element (i.e., time) that is unrelated to the health status of a individual. Measurement theory notifies that the TTO method cannot be classified as an accurate (unidimensional) measurement method for health states, because two distinct phenomena (health status, longevity) are measured simultaneously. In general, distortions of health-state values, if elicited with the TTO and the more traditional standard gamble technique, are widely recognized [37,38].

Several previous studies have investigated the relationship between health-state values derived from patients versus the general population. An overview article [6] identified nine study designs that have been used to study this issue. In general, the designs could be differentiated in terms of the type of health states, selection of study population, valuation task etc. Health states were divided into hypothetical states and actual states. Most studies compared patients' values for their own actual health state, as experienced at the time of measurement, with values for hypothetical health states pertaining to treatment outcomes or particular stages of disease [39-41]. In most cases, general population values were obtained by using an existing social tariff [42-45]. A few studies took an indirect approach to compare valuations for actual and hypothetical states [46]. Other studies analyzed values from different groups, values derived with different valuation techniques, or assessments of different conditions.

A research design that comes close to ours was used by Badia et al. [47]. In their study, 14 hypothetical EQ-5D health states were valued (EuroQol-VAS) by a sample of the general population and chronically ill patients. Their results show higher values from patients compared with the general population, especially for worse states. This difference persisted when controlling for age, gender, education level, health status, and self-rated health (See also: [48]). Their study design differed from ours in various ways. Their patient group was more heterogeneous, and patients did not assess their own EQ-5D description. A factor that may largely explain why they found large differences between patients and healthy people is that in their study the raw VAS scores have not been rescaled (e.g., calibrated to 0 = dead, 1 = full health). Unknowing assessment of the patient's own health state had been used earlier by Llewellyn-Thomas [41] for breast cancer. In this study patients' values for health states related to breast cancer scenarios were compared with the patients' actual stage of disease.

A potential limitation of our experimental study is the sample size of the patient groups. In particular, the group of rheumatoid arthritis patients was moderate in size. It was too small to allow us to use rank data as input for scaling models, e.g., Thurstone scaling [28] or extended rank-based models (e.g., discrete choice models), to arrive at aggregated metric (interval) values. Nevertheless, the mean statistics for the rank and VAS data show relatively small standard errors of the mean, and the mean values for the set of health states show a clear overall pattern. The interviewer may have influenced the obtained results from the patients, though we have no indication that this may have led to notable biases.

## Conclusions

The results of this study indicate that differences between patients and non-patients can be largely reduced and eventually eliminated if the deriving of health state values is worked out in a recognized measurement framework. Our findings also imply that instead of patients, people from the general population may be interviewed to quantify hypothetical health states. The only requirement is that the assessment of health states should take place under rigorous conditions. Essentially, this stipulates that a wide array of health states should be judged or assessed by simple comparative response tasks that are embedded in an established theoretical measurement framework.

## Competing interests

The authors declare that they have no competing interests.

## Authors' contributions

Conception and design: PFMK, NT. Provision of study materials and/or patients: PFMK, NT, TJMR, PLCMR. Collection and assembly of data: PFMK, NT. Data analysis and interpretation: PFMK, NT.  Manuscript writing: PFMK, NT, TJMR, PLCMR. Final approval of manuscript: PFMK, NT, TJMR, PLCMR. All authors read and approved the final manuscript.
